# Comparing Two Automated Techniques for the Primary Screening-Out of Urine Culture

**DOI:** 10.3389/fmed.2018.00353

**Published:** 2018-12-14

**Authors:** María Isabel Millán-Lou, Juan Manuel García-Lechuz, María Angeles Ruiz-Andrés, Concepción López, María José Aldea, P. Egido, María José Revillo, Antonio Rezusta

**Affiliations:** ^1^Servicio de Microbiología, Hospital Universitario Miguel-Servet, Zaragoza, Spain; ^2^IIS Aragón, Zaragoza, Spain; ^3^Department of Microbiología, Medicina Preventiva y Salud Pública, Universidad de Zaragoza, Zaragoza, Spain

**Keywords:** urinary tract infection, microbiological diagnostics, automated screening systems, UF-1000i, FUS200

## Abstract

Urinary tract infection is the most common human infection with a high morbidity. In primary care and hospital services, conventional urine culture is a key part of infection diagnosis but results take at least 24 h. Therefore, a rapid and reliable screening method is still needed to discard negative samples as quickly as possible and to reduce the laboratory workload. In this aspect, this study aims to compare the diagnostic performance between Sysmex UF-1000i and FUS200 systems in comparison to urine culture as the gold standard. From March to June 2016, 1,220 urine samples collected at the clinical microbiology laboratory of the “Miguel Servet” hospital were studied in parallel with both analysers, and some technical features were evaluated to select the ideal equipment. The most balanced cut-off values taking into account bacteria or leukocyte counts were 138 bacteria/μL or 119.8 leukocyte/μL for the UF-1000i (95.3% SE and 70.4% SP), and 5.7 bacteria/μL or 4.3 leukocyte/μL for the FUS200 (95.8% SE and 44.4% SP). The reduction of cultured plates was 37.4% with the FUS200 and 58.3% with the UF-1000i. This study shows that both techniques improve the workflow in the laboratory, but the UF-1000i has the highest specificity at any sensitivity and the FUS200 needs a shorter processing time.

## Introduction

Urinary tract infections (UTIs) are the most frequent infections diagnosed in hospitalized patients and primary care centres. They are associated with a high morbidity and cost (1–4). Therefore, urinalysis is one of the most important *in vitro* diagnostic tests in laboratory practice for the diagnosis of UTIs (2).

Urine culture and identification remain the gold standard procedures for definitive diagnosis of UTIs, but it is laborious, expensive, and has a response time of 24–48 h (5–9). Therefore, a rapid and reliable screening method would be useful to detect negative samples, avoiding costly and laborious culture procedures and reducing the total analysis time (8, 10). To avoid misclassification of positive urine samples, high sensitivity and negative predictive values are prerequisites for a screening method (1, 11). European Urinalysis Guidelines recommend an analytical sensitivity higher than 90–95% to detect asymptomatic bacteriuria at 10^5^ colony-forming unit/millilitre (CFU/mL) (12, 13).

UTIs screening is currently being carried out using automated and cost-effective diagnostic devices, including automated microscopy (IQ200), hybridization with fluorescent probes (Cellenium 160 US), measurement of bacterial ATP by enzymatic reaction (Coral UTI Screen), Flow Imaging Microscopy (FUS200), and flow cytometry (Sysmex UF-1000i) (6, 14). In our laboratory, we culture 300 urine samples every day and more than half of the plates are negative or contaminated. In terms of workload and costs, this means more than 2 h of faculty work and 2,000 euros per month. Previous research has reported a decrease in the number of samples cultured with highly sensitive screening systems such as flow cytometry (10). Automatic screening methods have been compared with other dipstick methods such as UriSed, Clinitek Atlas, Urisys 2,400, and Aution Max (3, 15, 16). However, only a few studies have compared different automated techniques for the screening of UTIs using bacterial and leukocyte counts (4, 17).

Geerts et al. (8) reported that the UF-1000i, developed to standardize sediment analysis in urine, rapidly quantifies urine particles, including leukocytes or white blood cells (WBCs), bacteria, red blood cells (RBCs), and casts by scattering and fluorescence. This instrument is a urine flow cytometer that utilizes a diode laser to quantify the sediment in two analytic channels using a fluorescent dye, which stains DNA. One of the channels analyses only the microbial content of the urine, while the other analyses RBCs, WBCs, casts, and other non-microbial sediments (18). After staining, the particles are transported to a flow cell and irradiated with a laser (λ 635 nm). On the other hand, Kocer et al. (5) described the FUS200 as an instrument whose measurement principles are based on flow imaging microscopy. This instrument is capable of detecting and counting settled particles using the sheath flow technique. Under the effect of a double layer sheath, the urine sample enters the flow cell in the form of a single cell layer. The FUS200 CCD camera captures 650 frames of images. All images are evaluated by high quality image processing software capable of detecting and classifying urine particles.

In this article, we compared two different fast-automated systems, the UF-1000i and the FUS200, to select the most suitable screening equipment to meet the needs of the laboratory. For this purpose, we employed the semi-quantitative urine culture as the reference method to analyse the capacity of both systems for the diagnosis of UTIs in a tertiary hospital.

## Materials and Methods

### Collection of Urine Specimens and Analysers

Between March and June 2016, 1,220 urine samples from inpatients and outpatients were analysed in the clinical microbiology laboratory at the “Miguel Servet” hospital, in Zaragoza, Spain. The sample size was determined using the PASS v13 software (NCSS Statistical Software) based on the Lin and Fine et al. method, using a 95% sensitivity and an accuracy of 5% for the prevalence UTI expected in our population. The Hospital Committee of Ethic approved this study (reference number: 07/2016).

All urine specimens included in this study were cultured and processed using the UF-1000i (Sysmex Corporation, Kobe, Japan) and FUS200 (Dirui Industrial CO, LTD, China) analysers within 24 h after collection, from Tuesday to Friday each week. Voided midstream urine was collected in tubes with boric acid. Samples were excluded from analysis if an inadequate sample volume (<8 mL) was provided or if excessive mucus, gross haemolysis or pyuria were observed on visual inspection to prevent blockage of the instrument or interference during measurement.

### Urine Culture

Prior to automated systems, 1 μL urine specimens were cultured using the WASP®DT: Walk*-*Away Specimen Processor (Copan Diagnostics, Murrieta, CA) on Brilliance UTI agar (Oxoid Ltd., Basingstoke, United Kingdom). The cultures, incubated at 35°C for 18–24 h, were considered as positive if growth ≥10^5^ CFUs/ml. We used MALDI-TOF analysis (MALDI Microflex LT, Bruker Daltonics, Bremen, Germany) to identify growing colonies. If there were three or more different types of colonies, the urine was considered as contaminated, although we classified it as negative for research purposes and it was not submitted to the identification procedure.

### Instrument Features

Some features to compare both systems and to select the best equipment were: general features (such as size, weight, electrical requirement, noise) and technical features (such as the number of samples processed per hour, consumables, noise level, limitations on the samples submitted, connectivity with the laboratory software, maintenance, cost).

### Data Analysis

Statistical analysis was performed using the SPSS ® 21.0 Statistical Packages (SPSS Inc., Chicago, Illinois, USA). In order to determine the best cut-off values, the receiver operating characteristic (ROC) curve technique was used for bacteria and WBCs. Sensitivity (SE), specificity (SP), positive predictive value (PPV), negative predictive value (NPV) and accuracy rate at the best cut-off values for bacteria and WBCs were also calculated considering the urine culture as the gold standard.

## Results

### Screening of Significant Bacteriuria

One thousand and two hundred and twenty (1,220) urine specimens were included, of which 213 (17.4%) were positive and 1,007 (82.6%) were negative. The majority of the specimens were collected from women (58.4%). Outpatients represented 53.3% (*n* = 650) and inpatients 46.7% (*n* = 570) of the subjects. The most common microorganisms identified were *Escherichia coli* (62%), *E. faecalis* (9.4%), *K. pneumoniae* (7%), coagulase-negative *Staphylococcus* (5.6%), *P. mirabilis* (3.3%), *E. cloacae* (2.8%), *P. aeruginosa* (2.8%), *S. agalactiae* (2.3%), *C. freundii* (0.9%), *K. oxytoca* (0.9%), *S. oralis* (0.9%), *E. aerogenes* (0.5%), *E. faecium* (0.5%), *P. vulgaris* (0.5%), and *C. albicans* (0.5%).

Ten samples were culture-positive but negative by the UF-1000i (false negatives, 0.8%) at a cut-off value of 138.8 bacteria/μL or 119.8 leukocyte/μL. The bacteria isolated from these ten samples were *P. mirabilis* (3); *C. albicans* (1); *P. vulgaris* (1); *E. faecalis* (1); *E. coli* (1); *P. aeruginosa* (1); *S. epidermidis* (1); and *S. agalactiae* (1). Nine false negative samples (0.7%) for the FUS200 were found at a cut-off value of 5.73 bacteria/μL or 4.3 leukocyte/μL. The bacteria isolated from these nine samples were *P. mirabilis* (3); *E. coli* (3); *P. vulgaris* (1); *E. faecalis* (1) and *S. agalactiae* (1). From the nineteen false negative samples, seven were identified by both methods: *P. mirabilis* (3); *P. vulgaris* (1); *E. faecalis* (1); *E. coli* (1), and *S. agalactiae* (1).

### Comparison of Diagnostic Accuracy and ROC Curve Analysis

**Figure 1:**
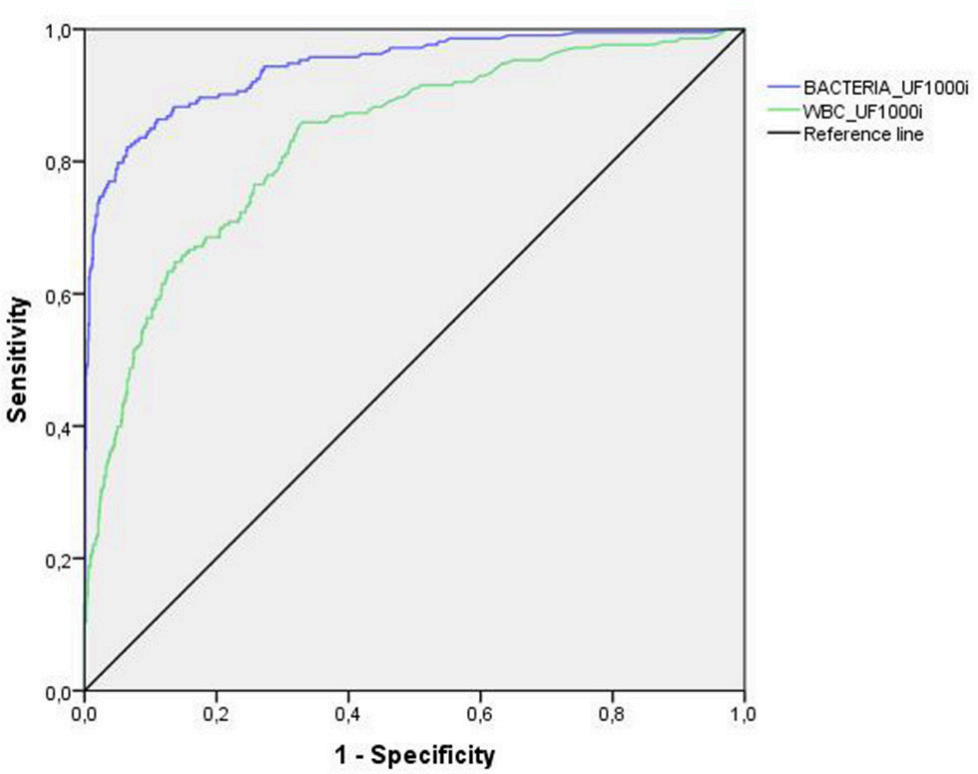
ROC curve analysis for the UF-1000i. Using flow cytometry, the AUC was 0.943 and 0.832 for bacterial and leukocyte count (>10^5^ CFU/mL), respectively.

**Figure 2:**
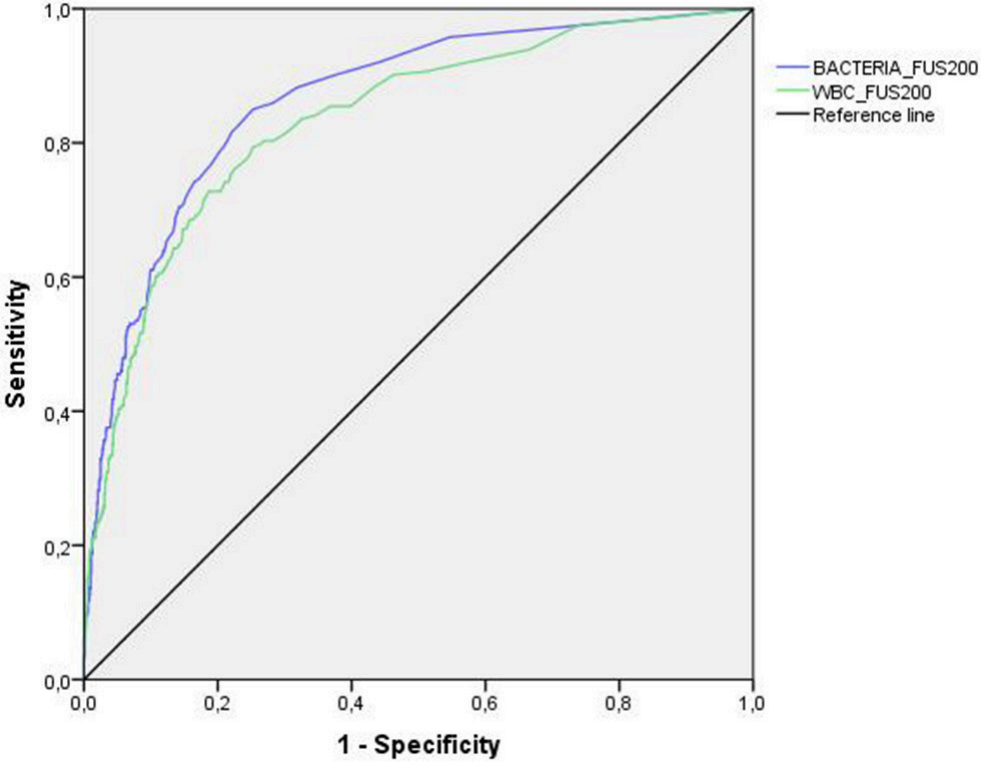
ROC curve analysis for the FUS200. Using flow microscopy, the AUC was 0.864 and 0.834 for bacterial and leukocyte count (>10^5^ CFU/mL), respectively.

Figures 1, 2 show the results of the analysis of the ROC curve carried out to assess the diagnostic value of bacteria and leukocyte counts of the two automated systems. In general, the area under the curve (AUC) for bacteria in the UF-1000i and the FUS200 performed better than the AUC for WBCs as a predictor of culture results. The sensitivity, specificity, NPV and PPV at different cut-off values for each method are shown in Table 1. We analysed the best combinations of bacterial and WBC counts as screening criteria to achieve the best sensitivity and specificity. The most balanced cut-off value for the UF-1000i and the FUS200 were 138 bacteria/μL or 119.8 leukocyte/μL, and 5.7 bacteria/μL or 4.3 leukocyte/μL, respectively. These results indicate a similar sensitivity of both systems; however, the UF-1000i showed a better specificity.

**Table 1:** Performance of the Sysmex® UF-1000i and the FUS200 at different cut-off thresholds for leukocyte and bacterial counts.

			**AUC**	**Cut-off value**	**Se (%)**	**Sp (%)**	**PPV (%)**	**NPV (%)**
UF-1000i	>10^5^*^^	Bacteria	0.943	89.4/μL	94.8	69.2	39.5	98.4
				255.3/μL	90.1	79.6	48.8	97.4
		WBC	0.832	3.8/μL	94.8	36.7	24.1	97.1
				6.3/μL	90.1	51	28	96.1
		Bac or WBC		138/μL−119.8/μL	95.3	70.4	40.5	98.6
				29.5/μL−154.3/μL	97.6	52.7	30.4	99.1
	>10^4^*^^	Bacteria	0.876	16.9/μL	95	42.6	31.1	96.9
				29.5/μL	90	54.3	34.9	95.2
		WBC	0.787	1.6/μL	95	13.7	23	91
				3.9/μL	90	37.7	28.2	93.3
FUS200	>10^5^*^^	Bacteria	0.864	1.4/μL	95.8	45.3	27	98.1
				2,86/μL	90.14	62.36	33.62	96.76
		WBC	0.834	2.1/μL	93.9	33.4	22.9	96.3
				4,3/μL	90.14	53.72	29.17	96.26
		Bac or WBC		5.7/μL−4.3/μL	95.8	44.4	26.7	98
				1.4/μL−4–3/μL	97.6	30	22.8	98.37
	>10^4^*^^	Bacteria	0.798	0.72/μL	93.49	29.72	26.57	94.37
				1.4/μL	88.50	45.35	30.59	93.54
		WBC	0.792	1.4/μL	93.87	25.96	25.65	93.96
				2.1/μL	90.04	33.79	27.01	92.57

* *Value obtained in culture (CFU/mL)*.

The application of cut-off values of 138 bacteria/μL or 119.8 leukocyte/μL for the UF-1000i and 5.7 bacteria/μL or 4.3 leukocyte/μL for the FUS200 would have led to reductions of 58.3 and 37.4% in the number of samples cultured, respectively. At the same level of SE and NPV, the UF-1000i had higher SP and PPV.

### Sample Processing Throughput

Significant differences were observed between the two automated methods in the number of samples processed per hour, i.e., the UF-1000i processed 66 samples/hour (100 samples/hour was processed in user's guide) and the FUS200 processed 120 samples/hour (120 samples/hour was processed in user's guide).

## Discussion

To perform rapid diagnostics of urinary tract infection, the use of automated devices to screen urine samples is an attractive option, and reduce the number of urine samples that will be cultured, reduce costs and decrease the amount of antibiotics prescribed unnecessarily (2, 10).

Bacterial counts of one or two microorganisms in the same culture have been classically considered as an important argument for the diagnosis or UTI. The presence of more than two microorganisms means an inadequate sampling procedure or a contaminated culture that in terms of our research was considered as a negative result in both screening systems. In contrast, there are studies that considered the contaminate culture as positive result or excluded from the studies these samples (6, 12, 19).

In our study, the AUC for bacteria was greater than the AUC for leukocytes in both systems. These results agree with those published by Broenen et al. (1) that showed a greater AUC for bacterial counts (0.96 vs. 0.79 CFU/mL) than for leukocyte counts. In addition, the corresponding sensitivity was higher for bacteria than for leukocytes considering any specificity. Our study shows greater specificity and sensitivity improvement for the combined count of bacterial and leukocyte than for bacterial count alone. However, some articles show not improvement of screening with combined counts than bacteria alone (19, 20).

According to the reviewed literature, the cut-off values used range from 25 to 230 bacteria/μL (2, 12, 18, 21–23). In our study, the best cut-off point was found after comparing the results of culture and bacterial or leukocyte count, and it was 138 bacteria/μL or 119.8 leukocyte/μL (SE 95.3%, SP 70.4%) with the UF-1000i (24). This cut-off value had a similar SE and a higher SP than the cut-off point obtained with the FUS200 (5.7 bacteria/μL or 4.3 leukocyte/μL; 95.8% SE, 44.4% SP) (Table 2).

**Table 2:** Diagnostic yield of the cut-off points obtained in published studies.

**Author/year**	**System**	**Cut-off point**	**Se/Sp**	**PPV/NPV**
Kocer (5)	FUS200	16 bac/μL; 34 WBC/μL	82.3%/58% 72.3%/65.2%	65%/77.5% 57.1%/78.5%
Yuksel (25)	FUS100	^*^ND for WBC	68%/89%	60%/92%
Manoni (21)	UF-1000i	>125 bac/μL; >40 WBC/μL	97%/94% 87%/79%	92%/98% 72%/92%
De Rosa (12)	UF-1000i	170 bac × 10^6^/L and 150 WBC × 10^6^/L	98.8%/76.5%	59.2%/99.5%
Jolkkonen (18)	UF-500i	Different cut-off points	93.4%/82.3%	^*^ND
Pieretti (22)	UF-1000i	65 bac/mL and 100 WBC/mL	98%/62%	53.7%/98.7%
Wang (23)	UF-1000i	bac>10^5^/mL or WBC >56/μL or yeast-like fungi >100/μL	86%/95%	91%/94%
van der Zwet (9)	UF-1000i	≥50 bac /μl and ≥20 WBC /μl ≥50 bac /μl or ≥20 WBC /μl	85%/79% 100%/54%	55%/95% 39%/100%
Broeren (1)	UF-1000i	230 bac /μl	95%/80%	^*^ND
Jiang (26)	UF-1000i	230 bac/ μl	54%/96%	56%/96%
Marschal (4)	UF-1000i	bac 3 × 10^4^/mL	80.9%/78%	^*^ND
Muñoz-algarra (11)	UF-1000i	50 bac/μL	91.3%/73.1%	67.6%/93.2%
March-Rossello (27)	UF-1000i	247,850 bac/mL or 31,800 WBC /mL	90.6%/66.3%	47.8%/95.4%
Gutiérrez-Fernández (14)	UF-1000i	690 bac/μL and 38 WBC/μL	92%/65%	39%/97%
Okada (28)	UF-50	^*^ND	83.1%/76.4%	62%/90.7%

* *ND: No Data*.

Few studies have compared different automated systems to screen for UTI using bacterial and leukocyte counts. Marschal et al. (4), compared the UroQuick, BACSYS-40i, and UF-1000i systems. Only the UroQuick showed less sensitivity (73%) but better specificity (92.8%) for the leukocyte count than the bacterial count (SE 80.9%, SP 78%). Shayanfar et al. (17) compared the Iris iQ200 and the UF-100. The Iris iQ200 showed less sensitivity for leukocyte and bacteria cut-off (76 and 85%) than the UF-100 (92 and 95%).

Our study shows that the cut-off point set for the UF-1000i and the FUS200 led to a reduction in the number of cultures of 58.3 and 37.4%, respectively. The reduction reported in other studies with the UF-1000i varies from 28 to 64.5% (1, 9, 18, 22, 27). The reduction for the FUS200 in our study (37.4%) was lower than the 65% found by Kocer et al. (5).

Automated systems provide the opportunity to analyse a large number of samples in a short time. In our study, the UF-1000i processed 66 samples per hour, and this is similar to the results published by Pieretti et al. (22). However, the FUS200 was faster and processed 120 samples per hour. We detected a difference between the time described in the UF-1000i user's manual and the actual processing time. The difference was due to the time spent to avoid carry-over. The UF-1000i has the option to configure additional rinse cycles when high counts are detected in a sample. Initially, this might be a disadvantage of the UF-1000i system together with its limitation in processing samples with excess mucus, gross haemolysis, or pyuria, but it guarantees no cross contamination between samples.

Another issue that affects the productivity of the laboratory is the maintenance time that will define the downtime for the system (29). We ensured that both systems need similar maintenance time. None of them can compete in terms of cost against culture (cheaper but arduous and time-consuming). A minimum difference of one cent of euro per sample between both systems cannot be an argument for their selection. The rest of the technical features verified, such as size, noise level, sample volume, homogenized sample rate and consumables, showed no significant differences between the UF-1000i and the FUS200 (data not shown). Another important concern is related with the quality of samples that they admit. The UF-1000i does not process samples with excess of mucus, gross haemolysis or pyuria, a limitation that was not found in the FUS200.

The selection of an ideal instrument for urine screening depends on the characteristics of the laboratory, the level of automation, the workload and efficient management (29). In this scenario, the cost of consumables and performance (number of samples per hour) will be of great importance for the decision-making process. The number of samples processed per hour should be enough to cover the daily peaks of growing activity (30).

In conclusion and based on the AUC, cut-off value, number of samples applied and maintenance time, our study shows that the UF-100i and the FUS200 can be appropriate in a microbiology laboratory, reducing unnecessary cultures and providing negative results in few hours. At equal high sensitivity level (95%) the UF-1000i showed higher specificity than the FUS200 instrument, but the FUS200 needs a shorter processing time.

## Author Contributions

AR, MM-L, and MR planned and designed the experiments. MM-L, MR-A, CL, PE, and MA performed the analyses. MM-L, JG-L, and AR wrote the paper.

### Conflict of Interest Statement

The authors declare that the research was conducted in the absence of any commercial or financial relationships that could be construed as a potential conflict of interest.
